# ADMM-TransNet: ADMM-Based Sparse-View CT Reconstruction Method Combining Convolution and Transformer Network

**DOI:** 10.3390/tomography11030023

**Published:** 2025-02-26

**Authors:** Sukai Wang, Xueqin Sun, Yu Li, Zhiqing Wei, Lina Guo, Yihong Li, Ping Chen, Xuan Li

**Affiliations:** 1 School of Computer Science and Technology, North University of China, Taiyuan 030051, China; wangsukai@nuc.edu.cn; 2 Shanxi Key Laboratory of Intelligent Detection Technology and Equipment, North University of China, Taiyuan 030051, China; sunxueqin@nuc.edu.cn (X.S.); 20240154@nuc.edu.cn (Y.L.); b20230507@st.nuc.edu.cn (Z.W.); guolina@nuc.edu.cn (L.G.); chenping@nuc.edu.cn (P.C.); 3 School of Information and Communication Engineering, North University of China, Taiyuan 030051, China; 4 Department of Mathematics, North University of China, Taiyuan 030051, China; 20230007@nuc.edu.cn

**Keywords:** sparse-view CT, CT reconstruction, ADMM, CNN, transformer

## Abstract

Background: X-ray computed tomography (CT) imaging technology provides high-precision anatomical visualization of patients and has become a standard modality in clinical diagnostics. A widely adopted strategy to mitigate radiation exposure is sparse-view scanning. However, traditional iterative approaches require manual design of regularization priors and laborious parameter tuning, while deep learning methods either heavily depend on large datasets or fail to capture global image correlations. Methods: Therefore, this paper proposes a combination of model-driven and data-driven methods, using the ADMM iterative algorithm framework to constrain the network to reduce its dependence on data samples and introducing the CNN and Transformer model to increase the ability to learn the global and local representation of images, further improving the accuracy of the reconstructed image. Results: The quantitative and qualitative results show the effectiveness of our method for sparse-view reconstruction compared with the current most advanced reconstruction algorithms, achieving a PSNR of 42.036 dB, SSIM of 0.979, and MAE of 0.011 at 32 views. Conclusions: The proposed algorithm has effective capability in sparse-view CT reconstruction. Compared with other deep learning algorithms, the proposed algorithm has better generalization and higher reconstruction accuracy.

## 1. Introduction

X-ray computed tomography (CT) provides high-precision volumetric imaging of anatomical structures within the human body and has established itself as a cornerstone in clinical diagnostics, offering non-invasive diagnostic capabilities with an exquisite spatial resolution [1]. However, the high radiation dose associated with CT scanning increases the risk of cancer in humans [2]. Radiation exposure to the human body follows the principle of a “dose–effect relationship”, meaning that the potential harm is directly related to the dose of radiation received. To minimize this risk, an As Low As Reasonably Achievable (ALARA) principle should be strictly adhered to. Under the premise of ensuring high-quality imaging, various technical measures should be employed to reduce radiation hazards to the lowest possible levels. One commonly adopted solution to reduce the radiation dose is to minimize the scanning angle by employing sparse-view scanning [3]. Nevertheless, the projection data obtained through sparse-view scanning is incomplete, and directly solving the inverse problem of the imaging model (e.g., using the filtered back projection (FBP) method [4]) leads to severe streaking artifacts in the reconstructed images. Consequently, the image quality often fails to meet the requirements for clinical diagnosis.

For the problem of sparse-view CT reconstruction, iterative reconstruction algorithms are commonly used (e.g., algebraic reconstruction technique (ART) [5], simultaneous algebraic reconstruction technique (SART) [6], and expectation maximization (EM) [7]). Compared to analytical reconstruction algorithms, iterative methods can improve reconstruction quality to a certain extent; however, satisfactory results remain challenging to achieve when the projection data are incomplete [8]. In recent years, regularized image reconstruction algorithms have been increasingly applied to address the issue of CT reconstruction with incomplete projection data. Regularized reconstruction methods based on a compressed-sensing (CS) theory leverage the sparse prior information of images [9], enabling accurate CT reconstruction from sparse-view data. However, regularized iterative reconstruction methods typically require the design of various regularization terms and the formulation of appropriate prior assumptions for different types of CT images. Additionally, these methods often involve cumbersome manual parameter tuning, making the adjustment process labor-intensive and highly challenging.

In recent years, with the development of deep learning technology [10,11], deep learning-based methods have been widely applied to CT reconstruction tasks [12]. By adopting data-driven modeling approaches, adaptive training of relevant parameters can be achieved. In particular, convolutional neural networks (CNNs) and Transformer networks have extensive applications and advantages in the field of CT imaging. Generally, deep learning-based CT reconstruction methods can be broadly categorized into image-domain-based reconstruction methods, domain-transformation-based reconstruction methods, and model-based plug-and-play reconstruction methods.

Image-domain-based methods leverage the powerful advantages of deep learning technology in computer vision applications, which have already been demonstrated in areas such as image denoising and high-resolution image reconstruction [13,14,15]. By employing well-established deep model networks as post-processing steps for images, these methods achieve high-quality reconstruction results. The U-net network [16] was initially one of the algorithms using convolution for semantic segmentation tasks and achieved excellent results in image segmentation. Subsequently, Jin et al. [17] applied the U-net network for CT reconstruction, combining the FBP (Filtered Back Projection) reconstruction algorithm with the U-net network, which can effectively eliminate the streak artifacts produced via FBP reconstruction. Zhang et al. [18] learned the initializer of the conjugate gradient algorithm, and the proposed network effectively reduced image noise while also reducing the number of training parameters and improving the network-training speed. Kang et al. [19] proposed an unsupervised model and designed a cycle-consistent adversarial denoising network, which has a better effect on noise suppression and detail preservation. Zhang [20] trained a Transformer-based neural network and decomposed the noisy low-dose CT image into high frequency and low frequency to extract content features and enhance low-dose CT image quality. Employing deep learning algorithms as a post-processing step for images can reduce computational costs to a certain extent. However, the features extracted by deep networks are highly influenced by the initial traditional reconstruction results. Especially when the projection data are incomplete and contains obvious noise, the initially reconstructed images contain relatively complex and obvious noise, which is difficult to eliminate even through deep network models. Moreover, the information lost during the initial reconstruction is also difficult to accurately recover through post-processing. Therefore, the above post-processing methods are more suitable for processing CT images with high reconstruction quality, low noise, and a small gap from the high-quality reconstruction results.

Domain transform methods refer to methods that directly map detector data to CT images. The networks can effectively extract implicit relationships within the data, which allows for the extraction and utilization of prior knowledge embedded within the data without the need to establish complex mathematical models for the distribution of the data. Zhu et al. [21] proposed the manifold-based reconstruction network AUTOMAP, defining image reconstruction as a data-driven supervised learning task that learns the mapping relationship between sensor and image domains. Compared to traditional reconstruction methods, both reconstruction artifacts and noise are reduced. He et al. [22] encoded the prior knowledge of transforming real object–projection geometric relationships into virtual relationships (depicted by under-sampled imaging geometry models) through feature extraction and representation learning. By combining under-sampling modeling strategies with prior information, this neural network is able to achieve rapid and accurate CT image reconstruction. For 3D CT reconstruction, Shen et al. [23] proposed the single-projection reconstruction algorithm, which enables 3D reconstruction from a single projection. Based on this method, Li et al. [24] trained an improved autoencoder network to learn shared structural features within three-dimensional volumes from two-dimensional projections, enabling the reconstruction of three-dimensional volumes from a single projection at any perspective to a specific perspective, reducing computational load while improving reconstruction accuracy. Sun et al. [25] used a deep network to parameterize a nonlinear mapping function from orthogonal projections to CT volumes for 3D reconstruction, further enhancing the reconstruction accuracy. Unlike traditional reconstruction algorithms, the aforementioned algorithm does not perform reconstruction through the mathematical inversion of the projection-imaging process. Instead, it reconstructs the target by utilizing the structural features extracted from the projections. However, the main limitation of the direct mapping from raw data to images is its severe dependence on large amounts of data and the high computational cost, especially the huge GPU memory requirement, which makes it difficult to be applied in practical systems.

The model-based plug-and-play reconstruction method integrates deep network models with iterative reconstruction algorithms. In the iterative process, the model incorporates a deep neural network as a prior term. Wu et al. [9] introduced an unsupervised K-sparse autoencoder (KSAE) as a prior term in the iterative model and trained it using the fidelity between the reconstructed image and the manifold as the optimization objective, demonstrating certain advantages in noise reduction and detail reconstruction in low-dose CT reconstruction; Gao [26] used a Markov Random Field-Texture (MRF-T) method to enhance the reconstruction details of local tissues in low-dose images by incorporating tissue textures extracted from full-dose images as prior knowledge; Han et al. [27] designed a deep network structure in the differential back-projection domain to perform Hilbert-related ill-posed deconvolution, effectively removing cone-beam artifacts by utilizing a data-driven inversion approach. Wu et al. [28] developed a unified optimized mathematical model that incorporates projection data and image prior knowledge into an analytical iterative framework, which is significantly effective in artifact removal. Zhang et al. [29] proposed a Deep Residual Iterative Minimization Network (DREAM-Net) based on a novel iterative reconstruction framework. Unlike conventional deep iterative reconstruction frameworks, DREAM-Net leverages deep neural networks for constraints in the projection domain, residual space, and image domain simultaneously. The model-based plug-and-play reconstruction method can be trained with a relatively small dataset and achieve good reconstruction quality. However, due to the lack of feedforward paths in the neural network in the algorithm, the computational cost is relatively high and it can only be applied to high-precision reconstruction scenarios with low requirements for reconstruction time.

Beyond conventional deep learning-based reconstruction techniques, current research encompasses projection-domain-based algorithms and dual-domain reconstruction frameworks that operate transversely across both image and projection domains. Notably, projection-domain reconstruction methods typically employ neural networks to refine incomplete projection data through task-specific feature enhancement, followed by image reconstruction from the augmented projections. Lee [30] completed missing projection information in the sinogram using a U-Net architecture, outperforming common interpolation methods and providing complete projection data for analytical reconstruction methods. Dual-domain reconstruction algorithms combine reconstruction methods from both the projection domain and the image domain, thereby further enhancing reconstruction accuracy. Li et al. [31] proposed a multi-domain joint learning framework that incorporates training modules in the projection domain, image domain, and residual domain, ensuring data consistency while significantly improving the detail information in the reconstructed images.

Deep learning-based sparse-view CT reconstruction algorithms have achieved some promising advancements, but these methods all have certain limitations. Image-domain-based methods as a step in post-processing can reduce some computational costs. However, when the projection data are highly incomplete, they can lead to excessive smoothing of the image. The main limitation of the domain transform method is its severe dependence on large amounts of data and the high computational cost. Furthermore, the networks are overly reliant on data-driven approaches, resulting in poor generalizability. In comparison, the model-based plug-and-play reconstruction method can be trained with smaller datasets and achieve good reconstruction quality, but the computational cost is high and can only be applied to high-precision reconstruction scenarios with lower requirements for reconstruction time. These algorithms, by expanding the iterative algorithm framework into distinct network layers, offer enhanced interpretability. Furthermore, being constrained by the optimization algorithm framework, they can effectively prevent overfitting, thereby possessing stronger generalization capabilities. However, most existing iterative unfolding methods are based on CNNs, which overlook the non-local correlations between images. To address this issue, this paper proposes a CT reconstruction algorithm that combines CNN and Transformer, expanding the receptive field range through self-attention mechanisms to enhance the model’s ability to learn global image representations. Additionally, the algorithm incorporates multi-head transposed attention model and gated feed-forward network to reduce computational complexity and to enhance the complementary image details between network layers.

The structure of the remainder of this paper is as outlined below: Section 2 details the implementation specifics of ADMM-TransNet. Section 3 presents experiments on simulated datasets, and conducts noise robustness and ablation experiments on the proposed method. Section 4 concludes with summaries and outlines future work.

## 2. Materials and Methods

In this work, we propose an ADMM-based iterative unfolding network for CT reconstruction that autonomously learns regularization priors and hyperparameters through a data-driven paradigm, thereby eliminating the necessity for manual prior design and reducing computational burden associated with parameter optimization while enhancing reconstruction fidelity. Furthermore, to address the limitation of CNN models in capturing non-local correlations within images, we propose ADMM-TransNet, the algorithm that integrates CNN and Transformer. By leveraging the ADMM iterative framework, this approach enhances the extraction of both local and global image features, preserves image details more effectively, and improves the accuracy of the reconstructed images.

### 2.1. Total Variation (TV) Method

The CT projection can be expressed mathematically in the form of a linear equation:

(1) Ax=y

In this context, *A* = {*a_ij_*} denotes the system matrix, *x* signifies the image vector to be determined, and *y* corresponds to the measured projection data acquired from detectors across multiple views. The objective of the reconstruction process is to deduce the unknown vector *x* utilizing the system matrix *A* and the projection data *y*.

Given a complete set of projections that are largely free from noise, Equation (1) can be inverted analytically using the FBP method, applicable to both fan-beam and cone-beam geometries. However, in scenarios where data are undersampled, Equation (1) admits an infinite number of solutions. The ART method and its variants can converge to the solution that is closest to an initial estimate. To achieve a plausible approximate solution, numerous optimization models incorporating regularization have been suggested. For the sake of brevity, the regularization reconstruction model can be formulated as follows:

(2) $$x=\underset{x}{\arg\;\min}E(x)=\underset{x}{\arg\;\min}\frac{1}{2}\left|\right|Ax-y|{|}_{2}^{2}+\lambda R(x)$$

where the notation $\left|\right|\cdot {\left|\right|}_{2}^{2}$ refers to the L_2_ norm. The initial term ensures data fidelity, aligning the reconstructed image vector *x* with the measured projection data *y*. The subsequent term serves as a regularization component, with *λ* being the regularization parameter that modulates the trade-off between the fidelity term and the regularization term.

In CT imaging, the image gradient approaches zero in homogeneous regions and becomes non-zero at the boundaries, resulting in a sparse gradient distribution. To capitalize on this sparsity, the L_1_ norm is applied to the gradient of the image, leading to the concept of Total Variation (TV) regularization. Employing the concept of anisotropic TV, the regularization term in Equation (2) can be articulated as follows:

(3) $$R(x)=||x|{|}_{TV}=\sum_{j}||D_{j}x|{|}_{1}$$

*D_j_* denotes the differential operator along the *j*-th direction. In two-dimensional (2D) scenarios, *D*_1_ and *D*_2_ correspond to the difference operators for the horizontal and vertical directions, respectively.

### 2.2. ADMM Algorithm for Optimized Model

The CT reconstruction model employing sparse regularization can be depicted as follows:

(4) $$x=\underset{x}{\arg\;\min}\frac{1}{2}{\left\|Ax-y\right\|}_{2}^{2}+\lambda {\left\|Dx\right\|}_{1}$$

Utilizing the Alternating Direction Method of Multipliers (ADMM) optimization technique, the objective function is effectively partitioned into several manageable sub-problems that are addressed sequentially. With the introduction of an auxiliary variable *z*, Equation (4) can be reformulated as follows:

(5) $$x=\underset{x}{\arg\;\min}\frac{1}{2}{\left\|Ax-y\right\|}_{2}^{2}+\lambda {\left\|z\right\|}_{1}\;\;\;\;s.t.\;z=Dx$$

In order to separate the variables *x*, *z*, and *α*, Equation (5) can be broken down into three distinct sub-problems:

(6) $$\left\{\begin{array}{l} \underset{x}{\min}\;\frac{1}{2}{\left\|Ax-y\right\|}_{2}^{2}+<\alpha ,Dx-z>+\frac{\rho }{2}{\left\|Dx-z\right\|}_{2}^{2}\;\\ \underset{z}{\min}\lambda {\left\|z\right\|}_{1}+<\alpha ,Dx-z>+\frac{\rho }{2}{\left\|Dx-z\right\|}_{2}^{2}\\ \underset{\alpha }{\min}<\alpha ,Dx-z> \end{array}\right.$$

The sub-problems associated with variables *x* and *α* can be iteratively solved using the gradient descent method. Regarding the *z* sub-problem, it can be directly resolved through the soft-thresholding approach [32]. The solution to the z sub-problem is given by the following:

(7) $$z=\max\{|Dx+\frac{\alpha }{\rho }|-\frac{\lambda }{\rho },0\}\times \mathrm{sgn}(Dx+\frac{\alpha }{\rho })$$

The sub-problems for *x* and α are addressed using the gradient descent method, with *β* defined as α/*ρ*. The optimization steps of the ADMM algorithm are as follows:

(8) $$\left\{\begin{array}{l} x^{\left(n\right)}=x^{\left(n-1\right)}-\eta \rho D^{T}(Dx^{\left(n-1\right)}+\beta^{\left(n-1\right)}-z^{\left(n-1\right)})-\eta A^{T}(Ax^{\left(n-1\right)}-y)\;\\ z^{\left(n\right)}=\max\{|Dx^{\left(n-1\right)}+\beta^{\left(n-1\right)}|-\frac{\lambda }{\rho },0\}\times \mathrm{sgn}(Dx^{\left(n-1\right)}+\beta^{\left(n-1\right)})\;\\ \beta^{\left(n\right)}=\beta^{\left(n-1\right)}+\gamma (Dx^{\left(n\right)}-z^{\left(n\right)}) \end{array}\right.$$

In the prior term, *D* and *D^T^* represent the different operators for the horizontal and vertical directions, respectively. To streamline the iterative process, the parameters in Equation (7) are consolidated by defining a new parameter that encapsulates the product of several parameters, which are adaptively learned throughout the iterations. Specifically, let *θ* = *η*×*ρ* and *ψ* = *λ*/*ρ*. The resulting iterative model is as follows:

(9) $$\left\{\begin{array}{l} x^{\left(n\right)}=x^{\left(n-1\right)}-\theta D^{T}(Dx^{\left(n-1\right)}+\beta^{\left(n-1\right)}-z^{\left(n-1\right)})-\eta A^{T}(Ax^{\left(n-1\right)}-y)\;\\ z^{\left(n\right)}=\max\{|Dx^{\left(n-1\right)}+\beta^{\left(n-1\right)}|-\psi ,0\}\times \mathrm{sgn}(Dx^{\left(n-1\right)}+\beta^{\left(n-1\right)})\;\\ \beta^{\left(n\right)}=\beta^{\left(n-1\right)}+\gamma (Dx^{\left(n\right)}-z^{\left(n\right)}) \end{array}\right.$$

### 2.3. Proposed ADMM-TransNet Network

**Figure 1:**
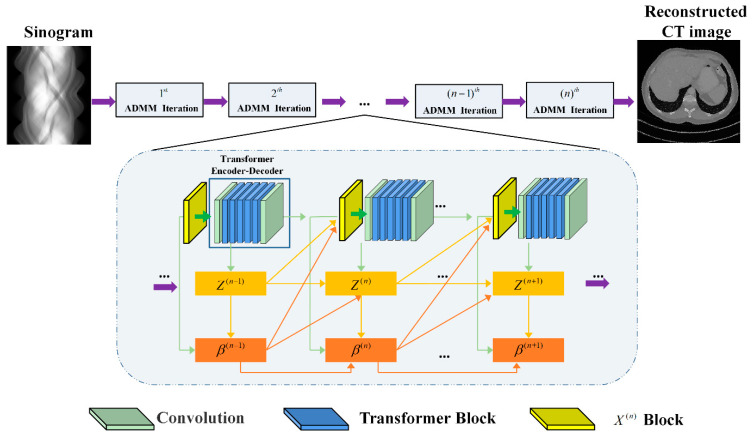
The overall structure of our proposed ADMM-TransNet.

Inspired by the modular structure of the ADMM algorithm and the plug-and-play strategy [33] that combines model-based inversion algorithms with advanced denoising algorithms, the network integrates an improved Transformer network and CNN into the reconstruction formula, using the network as a substitute for the prior term. The proposed ADMM-TransNet network embeds CNN-based modules (MDTA and GDFN) within the Transformer module under the framework of the ADMM algorithm, achieving a complementary integration of global and local information in images, thereby enhancing the overall performance of CT image reconstruction. Through model training, we obtain a sparsity variation that is more suitable for the training-sample images. By combining the Transformer with CNN, the network’s ability to learn both local and global features is enhanced, allowing for the reconstruction of higher quality CT images. The overall network structure is shown in Figure 1. The overall network structure is derived from the ADMM optimization Equation (9), with Figure 1 illustrating a deep network model of N layers (with *n* iterations), where each layer represents one iteration of the ADMM optimization algorithm. During the ADMM iteration process, three modules represent different optimization sub-problems, corresponding to the *x*(*n*) layer, *z*(*n*) layer, and *β*(*n*) layer, respectively. The prior information *D* and *D^T^* are embedded into the reconstruction formula using an improved Transformer network. The nth iteration of the ADMM algorithm corresponds to the nth layer of the network depth, where the projection data obtained from the measurements sequentially pass through the *x*(*n*) layer, *z*(*n*) layer, and *β*(*n*) layer. Ultimately, the *x*(*n*) layer outputs the final reconstructed image.

Utilizing the Transformer model to incorporate prior information in the ADMM algorithm enhances the reconstruction model’s ability to capture global features, while the CNN layers within the Transformer retain the capability to represent local image features. The integration of the Transformer model generally overcomes the limitations of CNN receptive fields; however, it is computationally intensive and may not be suitable for high-resolution image reconstruction tasks.

The pioneering work of applying the Transformer in the field of image processing is the Vision Transformer (ViT) model [34] published by Google. This work demonstrated that the Transformer could be directly applied to the domain of image processing. Subsequently, the Transformer model has achieved promising results in image classification, image detection, and image segmentation [35,36,37], marking a transition from convolution-based feature extractors to attention-based models. However, in the field of medical imaging, Transformer-based models have not been extensively studied, primarily due to the quadratic increase in computational complexity of the Self-Attention (SA) model with spatial resolution, making it challenging to apply in high-resolution medical images. To reduce computational demands, researchers have applied SA within a window size of 8 × 8 around each pixel [13] or by dividing the input image into non-overlapping blocks of size 48 × 48 and computing SA independently on each small sub-image [38]. However, these methods limit the spatial scope of SA, contradicting the goal of capturing global feature information. Therefore, this paper proposes modifications to the Transformer model by incorporating the Multi-Dconv head transposed attention and gated-Dconv feed-forward network modules [39] to reduce overall complexity.

**Figure 2:**
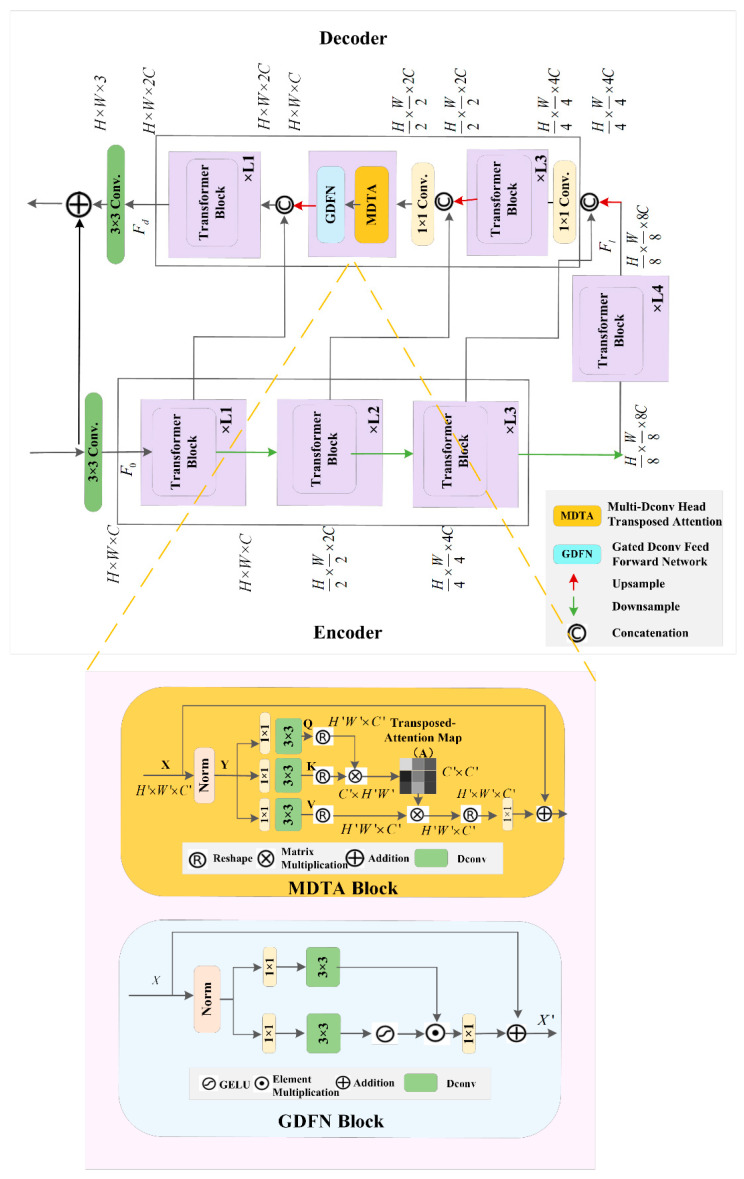
Architecture of the encoder–decoder Transformer.

The Transformer model, as shown in Figure 2, assumes a spatial dimension of H × W and a channel count of C for the input image $I\in R^{H\times W\times 3}$. During the encoding process, the dimensions are progressively reduced, while the number of channels is expanded. Initially, a convolutional operation is performed to obtain low-level feature embeddings with dimension $F_{0}\in R^{H\times W\times C}$. These shallow features are then processed through a four-layer encoder–decoder-structured Transformer module to yield deep features $F_{d}\in R^{H\times W\times 2C}$, with a gradual reduction in dimensions and an expansion in the number of channels. During decoding, the input features $F_{l}\in R^{\frac{H}{8}\times \frac{W}{8}\times 8C}$, and the image is restored layer by layer to a high-resolution image. Upsampling and downsampling within the network are carried out using pixel-unshuffle and pixel-shuffle [40], respectively. To enhance the image recovery process, features from the encoder are connected to those of the decoder through skip connections. Following the connection, the operation is a 1 × 1 convolution to reduce the channel count.

The MDTA module has linear computational complexity, with the key feature being the computation of SA over the image channels rather than individual image pixels. It introduces deep convolution to emphasize local features before calculating feature covariance to generate global features. Initially, the tensor $Y\in R^{H^{\prime }\times W^{\prime }\times C^{\prime }}$, after passing through a normalization layer, is processed by a 1 × 1 convolution to aggregate pixel-wise cross-channel context, followed by a 3 × 3 depthwise convolution to encode channel-wise spatial context, resulting in the projections for query (Q), key (K), and value (V):

(10) $$Q=W_{d}^{Q}{W_{p}}^{Q}Y$$

(11) $$K={W_{d}}^{K}W_{p}^{K}Y$$

(12) $$V={W_{d}}^{V}W_{p}^{V}Y$$

${W_{p}}^{\left(\cdot \right)}$ denotes a 1 × 1 convolution, and ${W_{d}}^{\left(\cdot \right)}$ represents a 3 × 3 depthwise convolution. Through reshaping and matrix multiplication operations, a transposed attention map *A* with dimension $R^{C^{\prime }\times C^{\prime }}$ is generated. The process can be expressed as follows:

(13) $$X^{\prime }=W_{p}Attention(Q^{\prime },K^{\prime },V^{\prime })+X$$

(14) $$Attention(Q^{\prime },K^{\prime },V^{\prime })=V^{\prime }Soft\max(K^{\prime }\times Q^{\prime }/\alpha )$$

*α* is a learnable scaling parameter.

The GDFN module is an improvement based on the traditional feed-forward network (FN). The structure of the GDFN is shown in the figure. The gating mechanism of the GDFN is beneficial for directing the network to focus on details that complement other levels. This gating mechanism is reflected in the network’s computation process as the element-wise product of two parallel linear transformation paths, where one path controls the output of the other through a GELU nonlinear activation [27]. Like the MDTA module, depthwise convolution is used to encode information from spatially adjacent pixel positions. For $X\in R^{H^{\prime }\times W^{\prime }\times C^{\prime }}$, the GDFN module can be expressed as follows:

(15) $$X^{\prime }=W_{p}^{0}Gating(X)+X$$

(16) $$Gating(X)=((\phi ({W_{d}}^{1}W_{p}^{1}(LN(X))\cdot ((W_{d}^{2}W_{p}^{2}(LN(X))$$

The activation function GELU is denoted by *φ*.

In the ADMM-TransNet network, the parameters involved in the Transformer model are denoted as Θ_T_, and the parameters in the reconstruction model are denoted as Θ_P_ = {*θ*, *η*, *ψ*, *γ*}. The set of training parameters is represented as Θ = {Θ_T_} ∪ {Θ_P_}. All parameters are optimized within the same framework using the Mean Squared Error (MSE) loss function to minimize the global loss function E(Θ):

(17) $$E(\Theta )=\frac{1}{N}\sum_{i=1}^{N}{\left\|x_{i}(\Theta )-x_{i}^{gt}\right\|}_{2}^{2}$$

where *x^gt^* corresponds to the ground truth of the image, and *N* is the number of image pairs used for training the network.

## 3. Experimental Steps

### 3.1. Traning Details

The ADMM-TransNet network uses the Adam algorithm [41] to optimize the loss function. During the training process of the network, the initial values of the parameters in the ADMM-TransNet iterative model are set as Θ_P_ = {*θ* = 2^−5^, *η* = 2^−5^, *ψ* = 2^−7^, *γ* = −10^−5^}. The initial learning rate of Θ_D_ is set to 10^−4^ and gradually decreases to 3 × 10^−6^, while the initial learning rate of Θ_p_ is set to 10^−8^ and slowly decreases to 3 × 10^−10^. ADMM-TransNet adopts a four-layer encoder–decoder architecture. From the first layer to the fourth layer, the number of Transformer Blocks is set to {4, 6, 6, 8}, and the number of attention heads in MDTA is {1, 4, 4, 8}. The number of channels is {48, 96, 96, 192}, and in the GDFN module, γ is set to 8/3. During training, if the validation loss remains unchanged for five epochs, the learning rate is reduced to half of its original value. The number of ADMM iterations (stages) is set to 20, and the initial value of x^(0)^ is set to the reconstruction result from the FBP algorithm. The training environment for this experiment is TensorFlow with a single Nvidia Tesla V100 32GB GPU, developed by NVIDIA Corporation in Santa Clara, CA, USA.

### 3.2. Dataset

To assess the performance of the ADMM-TransNet network, we employ the open-access dataset authorized by the “2016 NIH-AAPM-Mayo Clinic Low-Dose CT Grand Challenge” [42]. In total, 560 CT images from 10 cases were selected, 8 cases (448 CT images) were used for training, 1 case (56 CT images) for validation, and 1 case (56 CT images) for testing. The original projections in the dataset were obtained from helical trajectory CT scans and cannot be directly used for fan-beam CT image reconstruction; hence, the sparse view projections used in the experiments were simulated from the ground truth images. The ground truth reference images were acquired from the original data of conventional dose CT (NDCT) at 100 kV and downsampled to a size of 256 × 256. We performed forward projections of fan-beam scanning from the NDCT images for 32, 64, and 128 degree views. The specific simulated parameters are listed in Table 1.

### 3.3. Comparison Methods

Random projections were selected from the test dataset to conduct a comprehensive evaluation of the proposed reconstruction method. Six different methods were compared with the algorithm presented in this paper: FBP [4], ADMM [43], FBPConvNet [17], LEARN [44], ADMM-Svnet [45], and Trans-CT [20]. Among them, FBP and ADMM algorithms are two traditional CT reconstruction methods. The FBP algorithm is a widely used classical analytical reconstruction method, included in the paper to demonstrate that direct reconstruction under sparse view can lead to severe artifacts. ADMM is an iterative CT reconstruction method that employs ADMM to solve a CT model with constrained TV minimization. ADMM parameters: *μ* = 2^10^, *β* = 2^7^; Condition of convergence: number of iterations = 500. FBPConvNet, LEARN, ADMM-SVnet, and Trans-CT are deep learning-based CT reconstruction methods that have gained popularity in recent years. FBPConvNet is a CNN-based post-processing method for sparse-view CT, with a kernel size = 3; the initial learning rate was set to 10^−4^ and gradually decreased to 10^−6^, with the initial network input set to the FBP result. EARN is an iterative unfolding network based on CNN, using a one-step gradient descent method to solve a CT model with constrained minimization. The number of filters was set to 48; the kernel size was set to 5; the number of iterations was set to 50, and the initial network input was set to 0. The initial learning rate was set to 10^−4^ and gradually decreased to 10^−6^. ADMM-SVnet is our previous work, employing a CNN-based ADMM iterative unfolding network, with ADMM iteration stages set to 20, filter size = 3 × 3, number of filters = 32, and initial values for the iterative model parameters *η* = 2^−5^, *ψ* = 2^−7^, *φ* = 2^−7^, *γ* = −10^−5^. Trans-CT is a Transformer-based low-dose CT post-processing method, with the initial learning rate set to 10^−4^ and gradually decreased to 10^−5^.

## 4. Results

### 4.1. Simulation Data Research

In this work, we conducted a quantitative comparison of CT reconstruction results from different views (projections = 32, 64, 128) on the test dataset, with the results presented in Table 2. The MAE, PSNR, and SSIM values of the FBP reconstruction were the worst, indicating the poorest reconstruction quality. The performance metrics of ADMM were also unacceptable. Deep learning methods showed significantly better performance than traditional methods. ADMM-SVnet and LEARN demonstrated better performance than FBPConvnet and Trans-CT. Our proposed method achieves superior performance across all view counts, achieving a PSNR of 44.633 dB, SSIM of 0.996, and MAE of 0.006 at 128 views. Even under the most demanding 32-view condition, it consistently delivers robust and reliable metrics.

**Figure 3:**
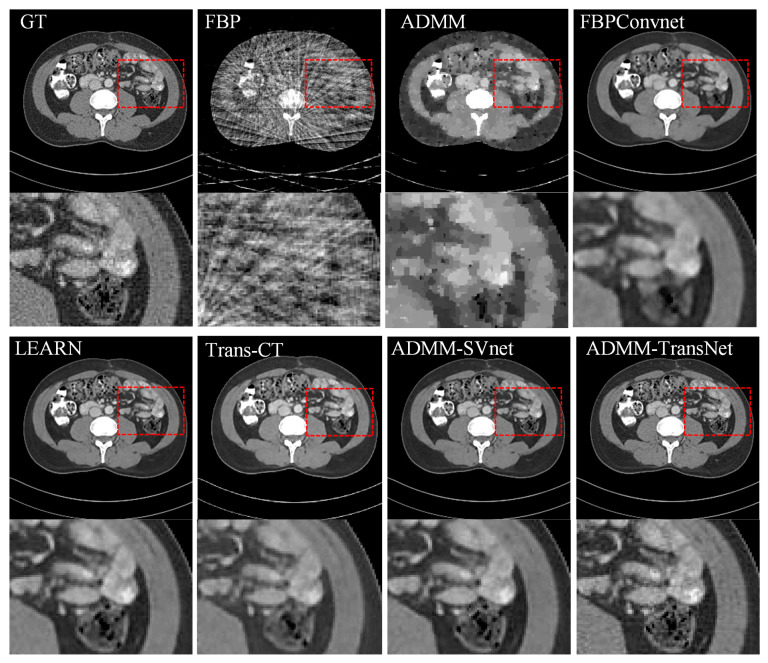
Comparative reconstruction results obtained from 32-view imaging data. The ground truth image is compared against FBP, ADMM, FBPConvnet, LEARN, Trans-CT, ADMM-SVnet, and ADMM-TransNet. The display window is [−150, 250] HU in size.

**Figure 4:**
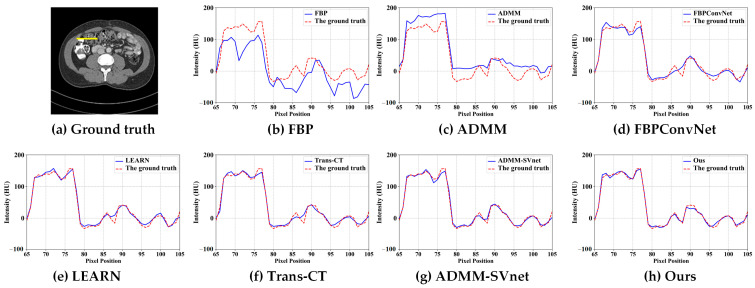
The intensity profiles along the yellow solid line in CT reconstructed images (from 32 views using (**b**) FBP, (**c**) ADMM (**d**) FBPConvNet, (**e**) LEARN, (**f**) Trans-CT (**g**) ADMM-SVnet, and (**h**) Ours).

**Figure 5:**
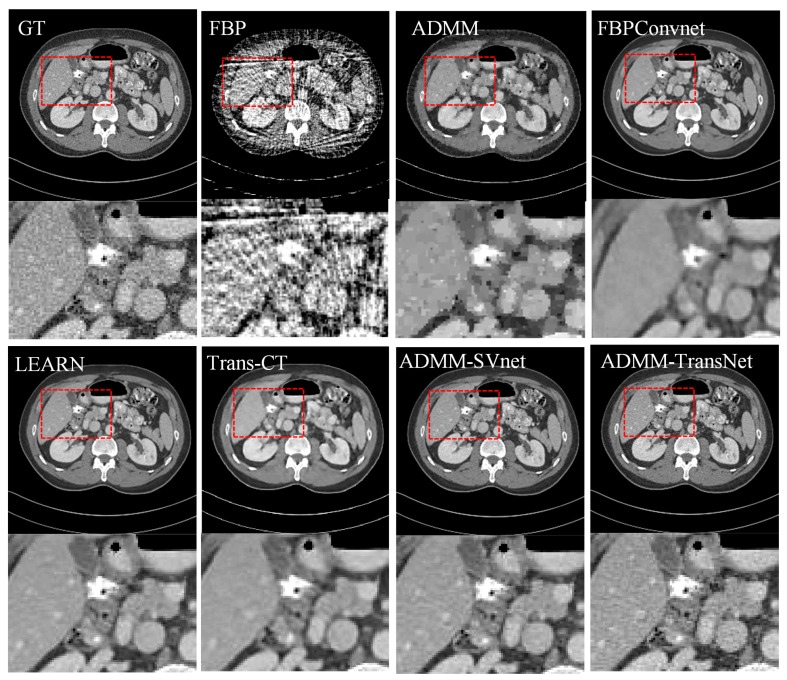
Comparative reconstruction results obtained from 64-view imaging data. The ground truth image is compared against FBP, ADMM, FBPConvnet, LEARN, Trans-CT, ADMM-SVnet, and ADMM-TransNet. The display window is [−150, 250] HU in size.

**Figure 6:**
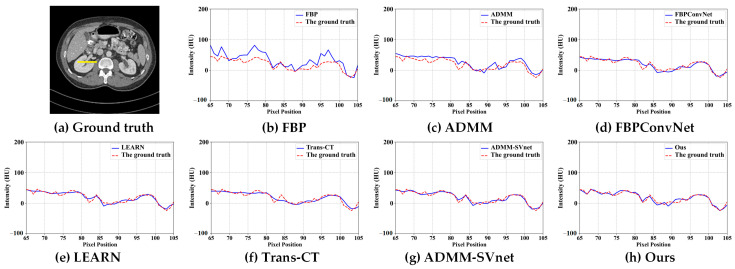
The intensity profiles along the yellow solid line in CT reconstructed images (from 64 views using (**b**) FBP, (**c**) ADMM (**d**) FBPConvNet, (**e**) LEARN, (**f**) Trans-CT (**g**) ADMM-SVnet, and (**h**) Ours).

**Figure 7:**
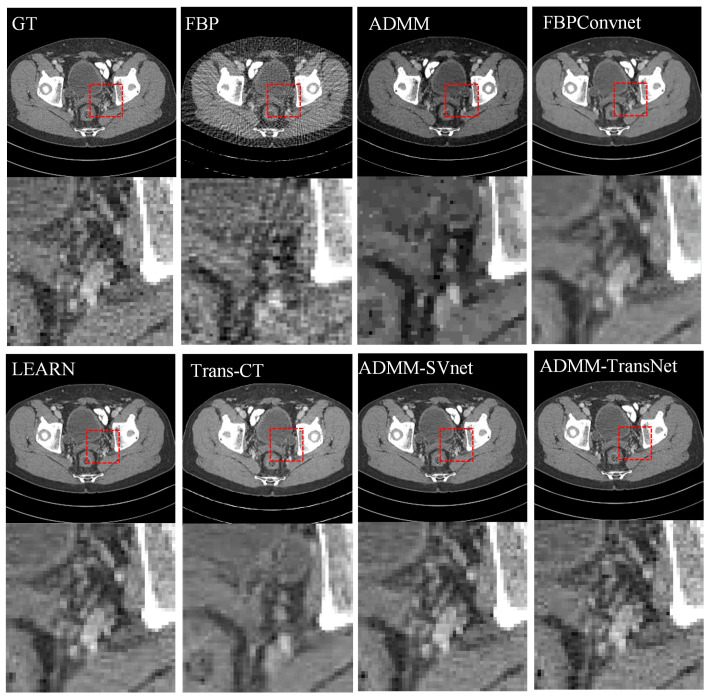
Comparative reconstruction results obtained from 128-view imaging data. The ground truth image is compared against FBP, ADMM, FBPConvnet, LEARN, Trans-CT, ADMM-SVnet, and ADMM-TransNet. The display window is [−200, 300] HU in size.

**Figure 8:**
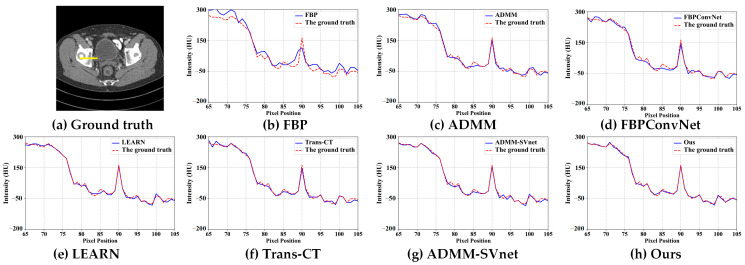
The intensity profiles along the yellow solid line in CT reconstructed images (from 128 views using (**b**) FBP, (**c**) ADMM (**d**) FBPConvNet, (**e**) LEARN, (**f**) Trans-CT (**g**) ADMM-SVnet, and (**h**) Ours).

In Figure 3, Figure 4, Figure 5, Figure 6, Figure 7 and Figure 8, we provide qualitative comparisons of CT reconstruction results across various sparse-view settings. The qualitative assessments align with the quantitative analyses, demonstrating that traditional CT reconstruction algorithms fail to generate satisfactory outcomes, as they are unable to accurately recover fundamental image structures. Conversely, deep learning-based methods have yielded significantly improved reconstruction results.

Specifically, Figure 3 illustrates the CT reconstruction results at 32 views. The FBP reconstruction exhibits excessive artifacts, disrupting image structures with streak-like artifacts. ADMM, a traditional regularized iterative reconstruction method, enhances image quality relative to FBP but fails to reveal organ structures adequately. FBPconvnet effectively mitigates most artifacts caused by sparse views yet struggles to recover detailed features, leading to overly smooth reconstructions. LEARN reconstruction yields comparatively superior results, revealing many detailed features. However, Trans-CT, while comparable to LEARN, still smooths out some fine details. ADMM-SVnet demonstrates a good overall reconstruction effect, preserving more image details. In contrast, ADMM-TransNet provides the best reconstruction results, being closest to the ground truth image and displaying the most detailed information. Figure 4 presents horizontal profiles at 32 views, where grayscale curves facilitate a more accurate comparison of the differences between algorithms. As shown in the figure, the image reconstructed by ADMM-TransNet is closer to the true value image.

Figure 4 and Figure 5 illustrate the reconstruction results at 64 views for various algorithms along with their corresponding horizontal profiles. As the number of projections increases, the quality of the reconstruction results improves significantly. However, FBP reconstruction continues to exhibit noticeable streak artifacts, and detailed information remains indistinct. While ADMM shows some improvement in edge reconstruction, the details are still challenging to discern. The FBPConvNet algorithm struggles with reconstructing low-contrast minute details. Additionally, LEARN, Trans-CT, and ADMM-SVnet demonstrate a loss of image features, with Trans-CT being particularly over-smoothed in detail representation. In contrast, our proposed reconstruction algorithm captures more detailed features, as evidenced by the grayscale curves in Figure 5, which show that images reconstructed by our method are closer to the ground truth.

Ultimately, experiments were conducted using projection data at 128 views, with the results presented in Figure 7 and Figure 8. It is evident that traditional algorithms, such as FBP and ADMM, continue to suffer from artifacts and oversmoothing issues, leading to unsatisfactory reconstruction outcomes. Among the deep learning algorithms, FBPConvNet exhibited limitations in capturing subtle details due to excessive smoothing. Conversely, the other four deep learning algorithms successfully reproduced fine image details. As illustrated in Figure 8, the grayscale curve comparison further highlights the performance differences among the algorithms. Specifically, the grayscale curves generated by the ADMM-TransNet algorithm demonstrate the highest consistency with ground truth images, thereby outperforming other methods in terms of reconstruction accuracy.

In summary, across datasets with 32, 64, and 128 views, the ADMM-TransNet reconstruction network introduced in this chapter effectively reconstructs fine image features often missed by other algorithms. The ADMM-TransNet algorithm significantly enhances the quality of reconstructed images, providing clearer details and edge information, thus achieving superior reconstruction results.

### 4.2. Model Structure Selection

**Figure 9:**
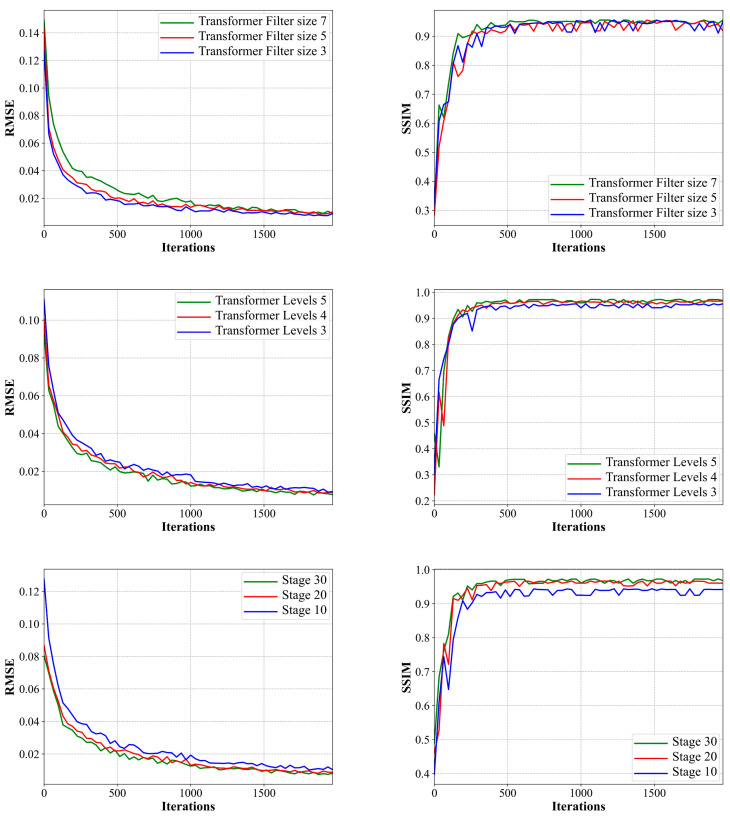
Model structure selection based on quantitative measures of the RMSE and SSIM as evaluated on the test dataset during training.

We evaluated the impact of key parameters in the ADMM-TransNet network on the evaluation metrics RMSE and SSIM, which include the size of the convolutional filters in the Transformer block, the depth of the Transformer network (Levels), and the number of ADMM iterations (stage). To assess the influence of the filter size in the Transformer block on reconstruction performance, this chapter set the filter size of the Transformer block as a variable, with the depth of the Transformer network (Levels) fixed at 4 (with the number of Transformer blocks set to 4, 6, 6, and 8 for Levels 1 to 4, respectively; the number of attention heads in the MDTA module set to 1, 4, 4, and 8; and the number of channels set to 48, 96, 96, and 192, respectively), and the number of ADMM iterations (stage) fixed at 10; to evaluate the impact of the depth of the Transformer network (Levels) on the network, this chapter set the filter size of the Transformer block to 3 and the number of ADMM iterations (stage) fixed at 10 in the experiments, with other parameters varying with the depth of the Transformer (Levels) detailed in Table 3. To assess the influence of the number of ADMM iterations (stage) on the reconstruction results, the depth of the Transformer network (Levels) was fixed at 4 in the experiments (with the number of Transformer blocks set to 4, 6, 6, and 8 for Levels 1 to 4, respectively; the number of attention heads in the MDTA module set to 1, 4, 4, and 8; and the number of channels set to 48, 96, 96, and 192, respectively), and the filter size of the Transformer block was 3. The experiments were conducted by optimizing the model on a training dataset with 32-view projections and then evaluating the performance on the corresponding test set, with the results shown in Figure 9.

(1) Impact of Filter Size in Transformer Block on Reconstruction Performance

We set the filter size in the Transformer block to 3 × 3, 5 × 5, and 7 × 7 to test the impact of different filter sizes on reconstruction performance. As can be seen from Figure 9, the RMSE performance gradually improves with the decrease in filter size but eventually tends to converge as the number of training epochs increases. The SSIM performance initially increases with the increase in filter size and also tends to converge with more training epochs. The smaller the size of the filter is, the better the performance of the model will be under the same number of iterations. Therefore, based on the above results, the final filter size in the Transformer block is set to 3 × 3.

(2) Impact of Transformer Network Depth (Levels) on Reconstruction Performance

To explore the impact of the depth of the Transformer network on reconstruction performance, this chapter sets the depth of the Transformer network to 3, 4, and 5 levels. The specific parameter settings involved in the Transformer network are listed in Table 2, Table 3 and Table 4. As shown in Figure 9, quantitative results improve with the increase in depth. However, deeper networks may lead to significant computational costs; therefore, to balance the network-training time and reconstruction performance, we set the depth of the Transformer network to 4.

(3) Impact of ADMM Iteration Count (Stage) on Reconstruction Performance

Lastly, we assessed the impact of the number of ADMM iterations (stage) on the network performance, as shown in Figure 9. We set the iteration count to 10, 20, and 30. It can be observed that both RMSE and SSIM performance gradually improve with an increase in the number of iterations. The change is more pronounced when the iteration count increases from 10 to 20, and while there is still an improvement from 20 to 30, the difference is not significant. Moreover, with the increase in ADMM iteration count, computational costs and processing time also gradually increase. Based on these results, to balance reconstruction performance and time costs, the number of ADMM iterations in the network is ultimately set to 20.

### 4.3. Noise Robustness Analysis

To verify the robustness of the proposed method against noise, we conducted reconstruction experiments on noise-corrupted projections within a dataset of 32 views. The noise was added according to the following formula [46]:

(18) $$noise_{i}=Poisson\{I_{0}e^{-y_{i}}\}+Normal(0,{\sigma_{e}}^{2})$$

*I*_0_ is the blank scan factor, and *y_i_* is the line integral of the attenuation coefficients along the *i*th ray. In our experiment, the blank scan factor *I*_0_ was set to 1 × 10^7^, 5 × 10^6^, 1 × 10^6^, 5 × 10^5^, and 1 × 10^5^. *σ*_e_^2^ is the variance in the electronic background noise.

**Figure 10:**
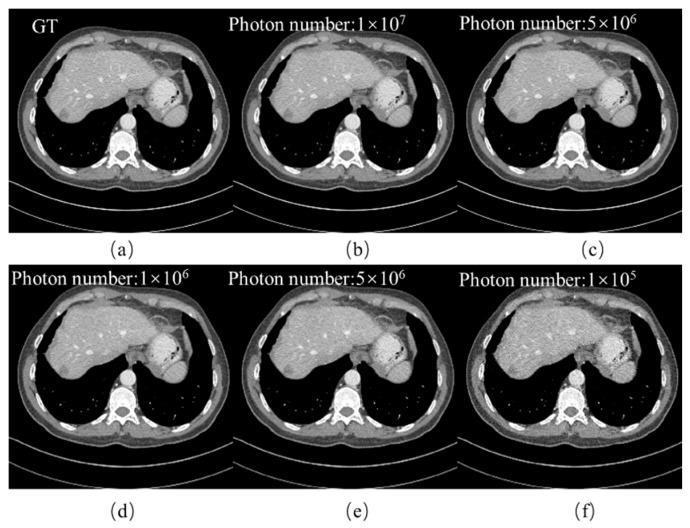
Reconstructed results (32-views) under different noise levels. (**a**) The ground truth image. From (**b**–**f**): *I*_0_ = 1 × 10^7^, *I*_0_ = 5 × 10^6^, *I*_0_ = 1 × 10^6^, *I*_0_ = 5 × 10^6^, and *I*_0_ = 1 × 10^5^. The display window is [−150, 250] HU in size.

**Figure 11:**
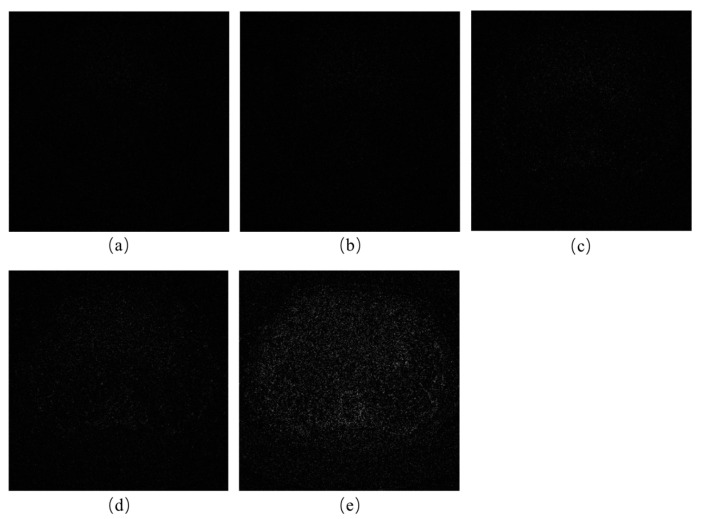
Absolute residuals between the reconstruction results and the reference image corresponding to Figure 10. From (**a**–**e**): I_0_ = 1 × 10^7^, I_0_ = 5 × 10^6^, I_0_ = 1 × 10^6^, I_0_ = 5 × 10^6^, and I_0_ = 1 × 10^5^.

Figure 10 demonstrates the reconstruction results of our method under various noise levels, and to further validate the aforementioned results, Figure 11 illustrates the absolute residuals between the reconstructed images and the ground truth images at different noise levels. To make the noise more apparent, the noise values were magnified by a factor of 2. It can be observed from the figures that the differences between the reconstructed results and the ground truth images are minimal. Table 4 presents the test results of the evaluation metrics RMSE, PSNR, and SSIM under different noise levels. These experimental results indicate that our algorithm can handle a wide range of noise levels and can accurately perform reconstruction across varying noise levels.

### 4.4. Ablation Experimental Study

To verify the effectiveness of the MDTA and GDFN module introduced in ADMM-TransNet, we conducted a series of ablation experiments on the proposed network using a dataset with 32 view projections, with the quantitative analysis of the experimental results presented in Table 5. The results indicate that the introduction of the MDTA module led to an improvement in the reconstruction results, with increases of 0.885 in PSNR and 0.019 in SSIM, respectively. That is, incorporating local information through deep convolution into the MDTA enhances the PSNR of the reconstruction results, thereby improving the robustness of the algorithm. The incorporation of the GDFN, compared to the traditional feedforward FN, resulted in improvements of 0.504 in PSNR and 0.013 in SSIM, endowing the proposed network with stronger noise resistance capabilities. Overall, the introduced MDTA module and GDFN module have contributed to the improvement of the network’s reconstruction performance.

### 4.5. Model Analysis

It is well known that Transformer-based methods face challenges in clinical application due to their extended training durations. To systematically evaluate computational efficiency, we conducted a comparative analysis of different model architectures as presented in Table 6. The baseline model was derived from the classic Vision Transformer (ViT) model [34], whose encoder architecture comprises alternating layers of multi-head self-attention (MSA) and Multilayer Perceptron (MLP). Specifically, this study evaluates the impact of the MDTA module and GDFN module on computational complexity and parameter quantity through component-wise comparisons. FLOPs (Floating-Point Operations) and inference time were calculated for 256 × 256 image processing, with ADMM network iterations set to 10 and the model employing a 4-layer encoder–decoder structure. The experimental results in Table 6 demonstrate that our proposed architectural innovations achieve substantial performance improvements while maintaining computational efficiency.

## 5. Discussion and Conclusions

In this study, we tackle the challenges associated with traditional sparse-view iterative models, which include difficulties in setting prior assumptions and the laborious manual parameter-tuning process. Additionally, deep neural networks exhibit a strong dependence on large datasets. To address these issues, we propose a CT reconstruction algorithm that integrates data-driven and model-driven approaches within an ADMM iterative framework. This algorithm leverages deep learning strategies to optimize prior terms and related hyperparameters in a supervised manner, thereby eliminating the need for manual design of prior information and reducing the burden of parameter tuning. Furthermore, by incorporating iterative model constraints, the proposed method mitigates the network’s reliance on extensive data samples, thus enhancing the robustness of sparse-view reconstruction algorithms. To improve upon CNN-based reconstruction algorithms that often overlook non-local feature correlations in images, we introduce a CT reconstruction algorithm that combines Transformers and CNNs. By utilizing the self-attention mechanism of the Transformer network model, we enhance the network’s ability to capture global image features. The introduction of the MDTA module and GDFN module optimizes the Transformer’s feature extraction process and computational efficiency, effectively reducing computational complexity while improving the fine structure and texture details of the reconstructed images. Finally, experimental results demonstrate the effectiveness of the proposed algorithm in terms of visual quality and quantitative metrics, showing that the CT reconstruction images obtained have more complete detail information and are robust against noise.

This study presents a deep learning-based framework for sparse-view CT reconstruction, addressing two critical challenges in conventional regularized reconstruction: optimal design of prior information and systematic selection of hyperparameters, while enhancing the model’s generalizability and representational capacity. Although the proposed method demonstrates improvements over existing approaches, several limitations require attention to enhance clinical applicability. Specifically, the current CT reconstruction network necessitates fixed scanning geometries and radiation dose levels during training, requiring separate network training for each parameter configuration. Future research should focus on developing modular architectures that enable simultaneous adaptation to variable scanning parameters and dose levels, coupled with establishing rigorous theoretical convergence guarantees for learned iterative reconstruction. Particular emphasis should be placed on investigating the interaction between data fidelity terms and neural-network-derived regularizers within the variational framework. These methodological advancements could bridge the critical gap between data-driven reconstruction techniques and clinical requirements while preserving interpretability essential for medical applications.

## Figures and Tables

**Table 1:** Data acquisition parameters.

	Parameters	Value
1	distance from the X-ray source to the flat-panel detector	1320.5 mm
2	distance from the X-ray source to the rotation center of the object	1050.5 mm
3	number of detectors	512
4	detector pixel size	0.127 mm
5	reconstruction size	256 × 256

**Table 2:** Quantitative results with different methods.

Views	128			64			32		
Metric	PSNR	SSIM	MAE	PSNR	SSIM	MAE	PSNR	SSIM	MAE
FBP	26.140	0.808	0.044	22.067	0.612	0.074	18.935	0.539	0.126
ADMM	33.751	0.929	0.021	30.883	0.915	0.027	29.753	0.907	0.038
FBPConvNet	39.854	0.952	0.020	34.243	0.938	0.028	30.648	0.916	0.035
LEARN	42.972	0.975	0.009	39.943	0.977	0.012	36.935	0.938	0.019
Trans-CT	40.877	0.966	0.011	35.855	0.941	0.019	32.430	0.922	0.023
ADMM-SVnet	43.229	0.995	0.007	42.974	0.989	0.008	40.212	0.972	0.013
Ours	44.633	0.996	0.006	43.726	0.992	0.007	42.036	0.979	0.011

**Table 3:** Model structure in ADMM-TransNet.

Levels		Transformer Blocks	Attention Heads in MDTA	Channels
3	L1	4	1	48
	L2	6	4	96
	L3	8	8	192
4	L1	4	1	48
	L2	6	4	96
	L3	6	4	96
	L4	8	8	192
5	L1	4	1	48
	L2	6	4	96
	L3	6	4	96
	L4	6	4	96
	L5	8	8	192

**Table 4:** Robustness analysis results for different noise levels.

Photon Number	1 × 10^5^	5 × 10^5^	1 × 10^6^	5 × 10^6^	1 × 10^7^
RMSE	0.011	0.008	0.008	0.008	0.008
PSNR	39.868	41.395	41.701	42.062	42.262
SSIM	0.979	0.986	0.987	0.987	0.988

**Table 5:** Quantitative evaluations for the ablation study.

Reconstruction networks	MDTA	MSA	√		√
	GDFN	FN		√	√
	PSNR	40.852	41.737	41.356	42.036
	SSIM	0.950	0.969	0.963	0.979

**Table 6:** Comparison results of trainable parameters and FLOPs.

Network	Cmoponent	FLOPs (G)	Params. (M)	Time (s)
Baseline	MSA + MLP	283.7	632.84	8.81
Multi-head attention	MDTA + FN	85.3	25.02	1.86
Feed-forward network	MTA + GDFN	86.3	25.12	1.86
Ours	MDTA + GDFN	87.7	25.31	1.87

## Data Availability

The datasets analyzed in this study are from “The 2016 NIH–AAPM–Mayo Clinic Low Dose CT Grand Challenge” and are available at: http://www.aapm.org/GrandChallenge/LowDoseCT/, accessed on 25 November 2024.
